# Discourse Analysis Uncovers Compulsory Altruism and Power Paradoxes for Family Caregivers of Those with Dementia

**DOI:** 10.1093/geroni/igab046.3381

**Published:** 2021-12-17

**Authors:** Candace Harrington

**Affiliations:** University of Louisville School of Nursing, Louisville, Kentucky, United States

## Abstract

Rural female family caregivers are under-represented, under-reported, and under-studied in rural caregiving and Alzheimer's disease-related dementias (ADRD) research. Caregivers' power struggles are often invisible and unknown. These constructs have social, policy, and practice implications for both family caregivers and their care recipients with ADRD. The purpose of this study was to explore how Foucauldian discourse analysis (FDA) elucidates rural female family caregivers' acquisition of caregiving roles for those with ADRD. FDA focuses on power structures and relationships in society as expressed through language and practices that affect marginalized groups. Textual data for this secondary analysis consisted of 157 pages of interview transcripts with 10 rural female caregivers. The systematic discourse analysis elucidated two socially constructed power and relationship structures. Compulsory altruism described complex socially constructed caregiving and gender role expectations, grounded in reciprocity, duty, and filial piety. A power paradox occurred when filial piety, duty, and reciprocity were in direct opposition to the caregivers' beliefs and value systems. In this sample, the subsequent sense of ambiguity about violating personhood and autonomy delayed black and white caregivers' responses to intervene with family members with ADRD. These delays resulted in near misses from wandering, driving-related accidents, cooking-related fires, financial exploitation by other family members, mistreatment that involved both caregiving dyad partners, and one tragic incident of a parent's death resulting from wandering-related exposure to elements. FDA was a valuable qualitative approach to elucidate family caregivers' power struggles that were previously invisible and unknown. These findings have broad implications for clinicians and researchers.

